# Primary chest wall sarcoma: advances in surgical management and outcomes

**DOI:** 10.1007/s00590-025-04260-1

**Published:** 2025-04-01

**Authors:** Shin Tanaka, Eiji Nakata, Tsuyoshi Ryuko, Takuto Itano, Yasuaki Tomioka, Kazuhiko Shien, Ken Suzawa, Kentaroh Miyoshi, Mikio Okazaki, Seiichiro Sugimoto, Toshifumi Ozaki, Shinichi Toyooka

**Affiliations:** 1 Department of General Thoracic Surgery and Breast and Endocrinological Surgery, Okayama University Graduate School of Medicine, Dentistry and Pharmaceutical Sciences, Okayama, Japan; 2 Department of Orthopedic Surgery, Okayama University Hospital, Okayama, Japan

**Keywords:** Primary chest wall sarcomas, Chest wall resection, Chondrosarcoma, Robust chest wall reconstruction

## Abstract

**Purpose:**

Although rare, primary chest wall sarcomas are complex malignancies necessitating optimal local control and comprehensive treatment. This study aimed to review 9 years of cases of primary chest wall sarcomas at a single institution, focusing on their histology, surgical management, and prognosis.

**Methods:**

A retrospective analysis was performed on 19 patients undergoing chest wall resection for sarcoma from 2012 to 2020. Data on demographics, tumor specifics, resection extent, and adjuvant therapies were collected. Surgical and postoperative outcomes were also assessed.

**Results:**

The median patient age was 64 years. Chondrosarcoma was the most common histology. R0 resection was achieved in all patients, with early postoperative complications occurring in 11% of the patients. Robust chest wall reconstruction was performed, resulting in minimal respiratory complications. The 5-year overall survival and disease-free survival rates were 94% and 68%, respectively. Tumor size and patient age were significant prognostic factors for local recurrence.

**Conclusion:**

Comprehensive surgical resection, coupled with multidisciplinary preoperative planning, achieves favorable outcomes. Patients aged ≥ 70 years and with tumor size ≥ 5 cm (*P* = .047) should be carefully followed up for local recurrence.

## Introduction

Primary chest wall sarcomas are rare and varied malignancies that grow slowly and innocuously to a relatively large size [1, 2]. To achieve optimal local control and long-term oncological outcomes, chemotherapy with or without radiation therapy (RT) is beneficial in patients with primary chest wall sarcomas, depending on the tumor histology. However, the pathological completeness of surgical resection represents the mainstay of treatment [3, 4]. The post-resection defects resulting from these tumors can be significant and often necessitate chest wall reconstruction, which may lead to postoperative complications [5–7]. The resection and reconstruction of chest wall sarcomas present unique challenges due to the area’s complex anatomy and functional significance. This study conducted a 9-year retrospective review of patients with chest wall sarcoma to examine common histological types, surgical management, and prognoses based on single-institution experiences.

## Materials and methods

### Overview

This was a retrospective observational study from a prospectively maintained database. This study was approved by the Institutional Review Board of our center (approval date: July 5, 2021; K2104-020). As per institutional protocol, prior informed and written consent was obtained from all the patients for future use of their data. A retrospective analysis was performed on a comprehensive database of patients having undergone chest wall resection for primary sarcoma at our institute from January 2012 to December 2020. Patients with at least 2 years of follow-up were included in this study. All patients had a confirmed pathological diagnosis and underwent surgical resection.

Patient demographic data (sex, age at diagnosis); tumor-specific data (date of diagnosis, primary site, histology, size, and grade); and the number of resected ribs, margins status, and treatment modalities received (neoadjuvant RT and/or chemotherapy or adjuvant RT and/or chemotherapy) were reviewed.

### Management protocol

#### Imaging

Computed tomography (CT) or 2-deoxy-2-(18F) fluoro-D-glucose positron emission tomography (PET) combined with CT (PET/CT) of the chest and abdomen were performed in all patients to determine the presence of distant metastasis upon initial presentation or at follow-up. We examined CT images of the chest, abdomen, and treated limb for all patients postoperatively while looking for distant metastasis every 3–4 months from the first to the third year of follow-up, twice a year until the fifth year, and then once a year thereafter for high-grade sarcomas. We followed up the patients every 6 months in the first to the third year and then once a year thereafter for low-grade sarcomas. Magnetic resonance imaging (MRI) was used to evaluate the signal intensity, size, and localization of the tumors.

### Diagnosis

Histological diagnosis was established according to the World Health Organization Classification of Tumors for all patients by expert pathologists. The grade was determined using the Fédération Nationale des Centres de Lutte contre le Cancer (FNCLCC) grading system, which is based on tumor differentiation, mitosis count, and tumor necrosis. Tumor size was defined as the maximal diameter on a cross sectional MRI examination. Pathological measurements were used to determine tumor size if pre-surgery cross sectional imaging was not available in patients with unplanned excision.

### Preoperative management

To enhance the mitigation of postoperative complications, our institution has established a specialized perioperative team, termed “perioperative management center (PERIO),” dedicated to comprehensive preoperative patient management. Although initially introduced and reported in the context of neurosurgery [8], this multidisciplinary system has since been widely implemented across various surgical specialties within our institution, including thoracic surgery. The PERIO system was employed in managing patients undergoing chest wall tumor resection in this study. This team comprises an array of multidisciplinary staff members to prevent perioperative complications and to improve the postoperative course. The PERIO system enhances functional recovery after surgery by consistent rehabilitation.

All patients underwent thorough evaluation within a multidisciplinary tumor board prior to the commencement of any treatment. Throughout the analyzed timeframe, the patients received treatment from a proficient multidisciplinary team of physicians. In cases where surgery was deemed necessary, a specialized team consisting of plastic, respiratory, and orthopedic surgeons deliberated and determined the optimal extent of tumor resection and the most suitable method for reconstructing the resultant defect.

### Treatment

Our chest wall sarcoma treatment strategy involves R0 resection in all cases. Preoperative planning is crucial, particularly when tumors are near major nerves or vessels, to ensure adequate margins. We routinely use MRIs with gadolinium-enhanced, fat-saturated T1-weighted sequences in axial and sagittal sections to plan resection margins and evaluate tumor growth patterns. Surgical excision margins are based on the American Joint Committee on Cancer residual tumor classification (R classification): R0 indicates no malignancy, R1 shows microscopic tumor cells at the inked border, and R2 signifies grossly positive margins. We aimed to resect tumors with a 2-cm margin to achieve R0 resection.

### Reconstructions

Following radical resection of chest wall sarcoma, a wide defect of the chest wall is inevitable. To restore structural integrity and safeguard intrathoracic organs, we primarily performed chest wall reconstruction using two sheets of polypropylene (Prolene; Ethicon, Somerville, NJ, USA). Given the directional elasticity inherent in Prolene polypropylene mesh, we arranged the two sheets orthogonally to each other, thereby imparting stability and flexibility essential for optimal functional outcomes [9]. The sandwich technique, which involves Prolene mesh with methyl methacrylate inside, was not used in our study. This is because our standard dual-layer Prolene mesh reconstruction has consistently provided adequate structural support without notable complications, eliminating the need for additional reinforcement.

### Adjuvant and neoadjuvant therapies

Conventional external beam RT was administered preoperatively when the tumor was close to the major nerves or vessels and had difficulty obtaining an efficient margin. In this study, all cases achieved R0 resection, and no patients required postoperative RT. Preoperative RT was administered to one patient. The radiation dose was 45–60 Gy with boost RT. Chemotherapy was administered to three patients: one patient with Ewing sarcoma received both neoadjuvant and adjuvant therapy, while the other two patients received only adjuvant therapy.

### Postoperative complications

All postoperative surgical complications were graded according to the Clavien–Dindo classification (CDC). This defines five grades of severity (grades I, II, IIIa, IIIb, IVa, IVb, and V). We especially analyzed wound infection, wound dehiscence, skin necrosis, postoperative hemorrhage, and wound pain. The numeric rating scale (NRS) was used to assess postoperative pain. NRS scores were compared at rest and during movement on the first postoperative day (POD) and seventh POD. Oxygenation levels were compared using preoperative SpO2 levels and levels on the first and seventh PODs.

### Assessment of study outcomes

We investigated the occurrence of complications and determined the association between the occurrence of complications and the following variables: age, sex, histology, tumor size, surgical margin, unplanned excision, receipt of chemotherapy, and RT. We analyzed local recurrence-free survival (LRFS), metastasis-free survival (MFS), and overall survival (OS). LRFS was calculated from the date of surgery to the date of local recurrence or to the date of the last follow-up. Those who died without recurrence or metastases were censored on the date of their death. MFS was calculated from the date of diagnosis to the date of metastasis diagnosis or the last follow-up. OS was calculated from the date of diagnosis to the date of death or the last follow-up visit. Survival rates were estimated using the Kaplan–Meier method. The Mann–Whitney U test was used to analyze continuous parameters, whereas Fisher’s exact test was used for categorical parameters. Logistic regression was used for multivariate analysis. Survival curves were plotted using the Kaplan–Meier method. In this analysis, a *p*-value of < 0.05 was considered statistically significant.

## Results

### Patient characteristics and tumor histology

Between 2012 and 2020, 19 patients received surgical treatment for primary chest wall sarcoma. The patients’ median age was 64 years, and most patients were male (58%) (Table 1). Among the patients, malignant bone tumors were 53%, and malignant soft tissue tumors were 47% of all the sarcoma cases. Chondrosarcoma was the most prevalent diagnosis, accounting for nine cases, followed by liposarcoma and myxofibrosarcoma in three cases each, undifferentiated sarcoma in two cases, and one case each for Ewing sarcoma and synovial sarcoma. Tumor sizes varied, with a median (range) of 4.8 (2–20) cm. Ten patients had tumors measuring < 5 cm, and nine patients had tumors ≥ 5 cm.

**Table 1:** Patient, tumor and surgical procedure characteristics

Variable	Value
Age (years)	64 (16–84)*
*Sex*	
Male	11 (58%)
Female	8 (42%)
*Diagnosis*	
Chondrosarcoma	9 (47%)
Liposarcoma	3 (16%)
Myxofibrosarcoma	3 (16%)
Undifferentiated sarcoma	2 (11%)
Ewing sarcoma	1 (5%)
Synovial sarcoma	1 (5%)
*Histological grade (FNCLCC)*	
Low (Grade 1)	4 (21%)
Intermediate (Grade 2)	11 (58%)
High (Grade 3)	4 (21%)
*Tumor location (Bone; n* = *10)*	
Rib	7 (70%)
Sternum	3 (30%)
Tumor size (cm)	4.8 (2–20)*
*Chemotherapy*	
Neoadjuvant	1 (5%)
Adjuvant	3 (15%)
*Radiotherapy*	
Preoperative	1 (5%)
Postoperative	0 (0%)
*Resected tissue*	
Rib only	15 (79%)
Sternum and ribs	2 (11%)
Sternum and diagram	1 (5%)
Number of rib resection	3 (0–6)*
*Resection margins*	
R0	19 (100%)
R1	0 (0%)
R2	0 (0%)
*Reconstruction*	
No	4 (21%)
Mesh only	12 (53%)
Muscle flap only	1 (5%)
Mesh and muscle flap	2 (11%)
*Postoperative complications Clavien–Dindo classification*	
I	10 (53%)
II	2 (11%)
IIIa	2 (11%)
IIIb	1 (5%)
IVa	0 (0%)
IVb	0 (0%)
V	0 (0%)

^*^Data are presented as median (range)

*FNCLCC* Fédération Nationale des Centres de Lutte contre le Cancer

Histopathological analysis according to the FNCLCC grading system revealed that tumors were grade 1 in 4 patients, grade 2 in 11, and grade 3 in 4.

### Extent of resection

We performed 18 (95%) lateral chest wall resections and 4 (21%) sternectomies. Three cases overlapped because the sternum and ribs were resected. The median number of rib resections was three (range 0–6). Partial sternectomy (defined as resection of < 90% of longitudinal diameter) was performed in three patients: lesions at the middle third underwent resection of the body and preservation of the manubrium and xiphoid process (*n* = 2), and lesions at the upper third underwent resection of the body and preservation of the manubrium of the xiphoid process (*n* = 1). A subtotal sternectomy, defined as resection of 90% of the longitudinal diameter of the sternum, was performed in one case for large lesions of the sternal body, sparing the xiphoid and upper manubrium. A total sternectomy was not performed. Resections were extended to the diaphragm in one, the pectoralis major muscle in three, and the serratus anterior muscle in one patient. Appropriate selection of these techniques achieved R0 resection in all patients (Table 2).

**Table 2:** Risk factors for local recurrence and distant metastases

Variable	Category	Patients, *n*		*P*-value
		Patients with	Patients without	
		local recurrence	local recurrence	
*Risk factors of local recurrence*				
Age, years	< 70	0	11	0.027
	≥ 70	3	5	
Sex	Male	1	10	0.35
	Female	2	6	
Histology	Chondrosarcoma	0	8	0.11
	Others	3	8	
Tumor size	< 5 cm	0	10	0.047
	≥ 5 cm	3	6	
FNCLCC grade	Grade 1	0	4	0.75
	Grades 2, 3	3	12	
Chest wall resection	< 3 ribs	1	7	0.74
	≥ 3 ribs	2	9	
Chemotherapy	Yes	1	2	0.36
	No	2	14	
Radiotherapy	Yes	0	1	0.66
	No	3	15	
*Risk factors of distant metastases*				
Age, years	< 70	0	11	0.08
	≥ 70	2	6	
Sex	Male	0	11	0.08
	Female	2	6	
Histology	Chondrosarcoma	1	10	0.81
	Others	1	7	
Tumor size	< 5 cm	0	10	0.12
	≥ 5 cm	2	7	
FNCLCC grade	Grade 1	1	3	0.33
	Grades 2, 3	1	14	
Chest wall resection	< 3 ribs	1	7	0.81
	≥ 3 ribs	1	10	
Chemotherapy	Yes	1	2	0.16
	No	1	15	
Radiotherapy	Yes	0	1	0.72
	No	2	16	

*FNCLCC* Fédération Nationale des Centres de Lutte contre le Cancer

### Chest wall reconstructions

Chest wall reconstruction was performed utilizing prosthetic materials in 13 (68%) patients, with 11 receiving dual sheets of polypropylene mesh for the reconstruction. Among the four sternal reconstructions, a monofilament polypropylene mesh (Bard® Mesh; C.R. Bard, Inc., Murray Hill, NJ, USA) and an e-PTFE mesh (Gore-Tex Dual Mesh®, W. L. Gore and Associates, Inc., Flagstaff, AZ, USA) were used in two instances each (Table 2). The remaining two sternal reconstructions were performed using the two-sheet method. Additionally, a muscular flap was incorporated in three (16%) patients.

### Postoperative complications

**Fig. 1:**
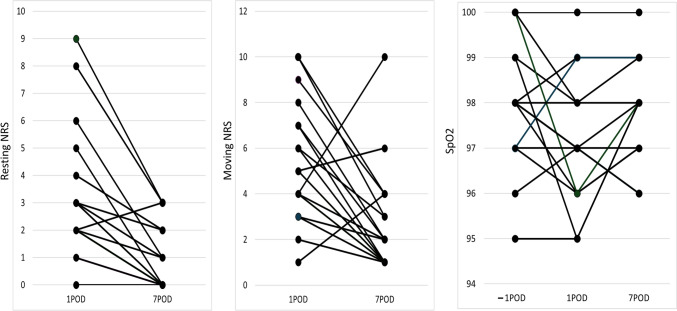
Comparison of pain at rest and pain with movement (numerical rating scale, NRS) on postoperative days (POD) 1 and 7, and the changes in oxygenation values from preoperative to POD 7

Early postoperative complications (within 30 days after surgery) above CDC IIIa occurred in two (11%) patients. These complications were associated with flap reconstruction in two patients. Partial necrosis of the flap occurred in one patient, whereas one with a free flap was healed by debridement. Late postoperative complication above CDC IIIa developed in one case of complicated resection of the diaphragm. Two months post-surgery, the patient developed a diaphragmatic hernia and underwent partial resection of the strangulated small intestine along with diaphragmatic reconstruction. The median resting NRS score for pain was 3 on the first POD and decreased to 0 by the seventh POD, representing a significant decrease over time (*P* < 0.001). The median NRS score for pain during movement was 5 on the first POD and decreased to 2 by the seventh POD, representing a significant decrease over time (*P* < 0.001) (Fig. 1). Flail chest, a typical complication following chest wall reconstruction, and the associated decline in respiratory function and activities of daily living were not observed in any patients. Additionally, there were no perioperative deaths within 30 days after surgery.

### Adjuvant and neoadjuvant therapies

In total, three (16%) patients received adjuvant therapy. One (5%) patient with Ewing sarcoma received both neoadjuvant and adjuvant chemotherapy, along with preoperative radiotherapy. Additionally, two (11%) other patients received adjuvant chemotherapy. No other patients underwent neoadjuvant chemotherapy or radiotherapy, and no patients received postoperative radiotherapy.

### Long-term outcomes

**Fig. 2:**
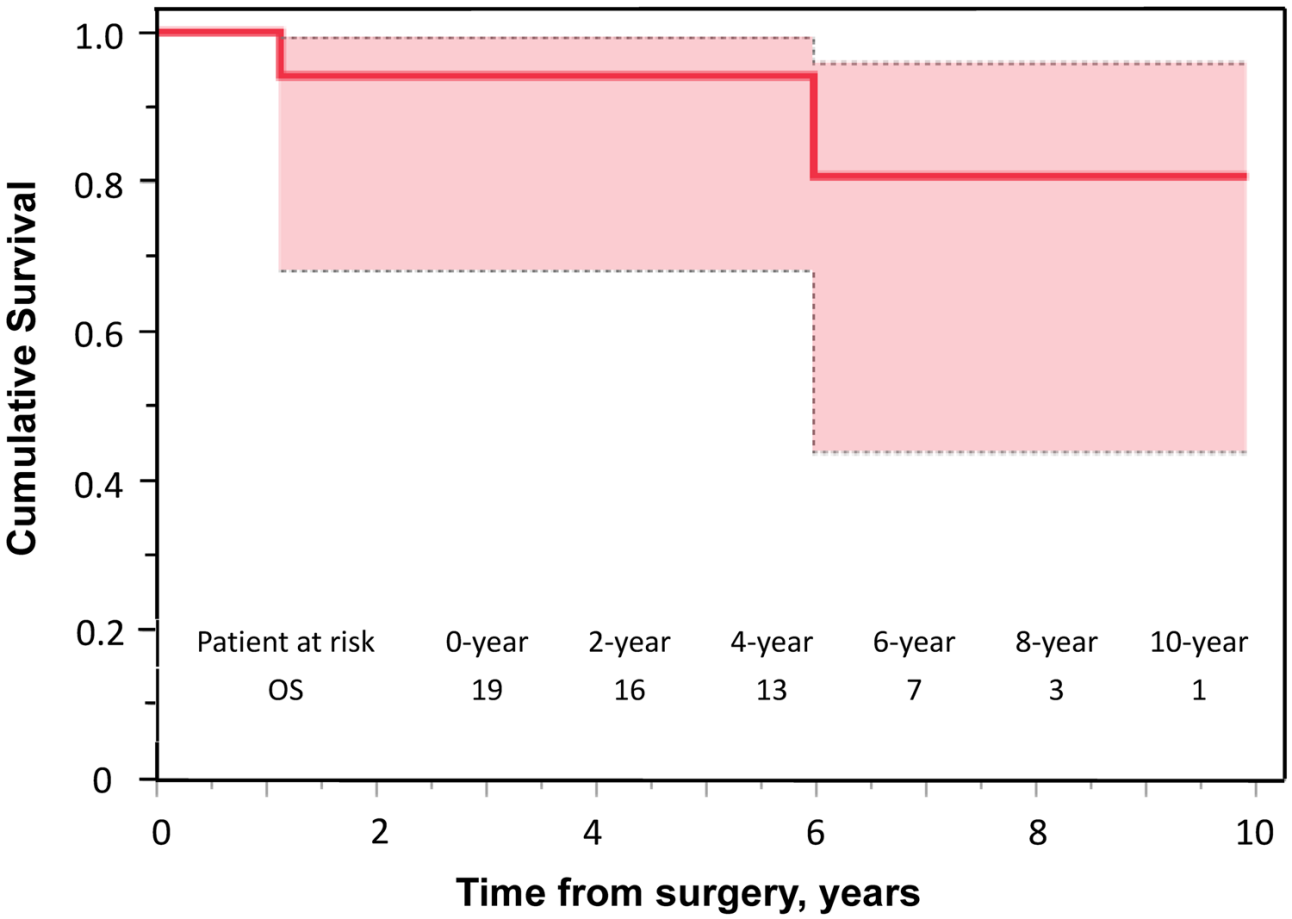
Kaplan–Meier estimates for the overall survival curve of 19 patients with primary chest wall sarcoma

**Fig. 3:**
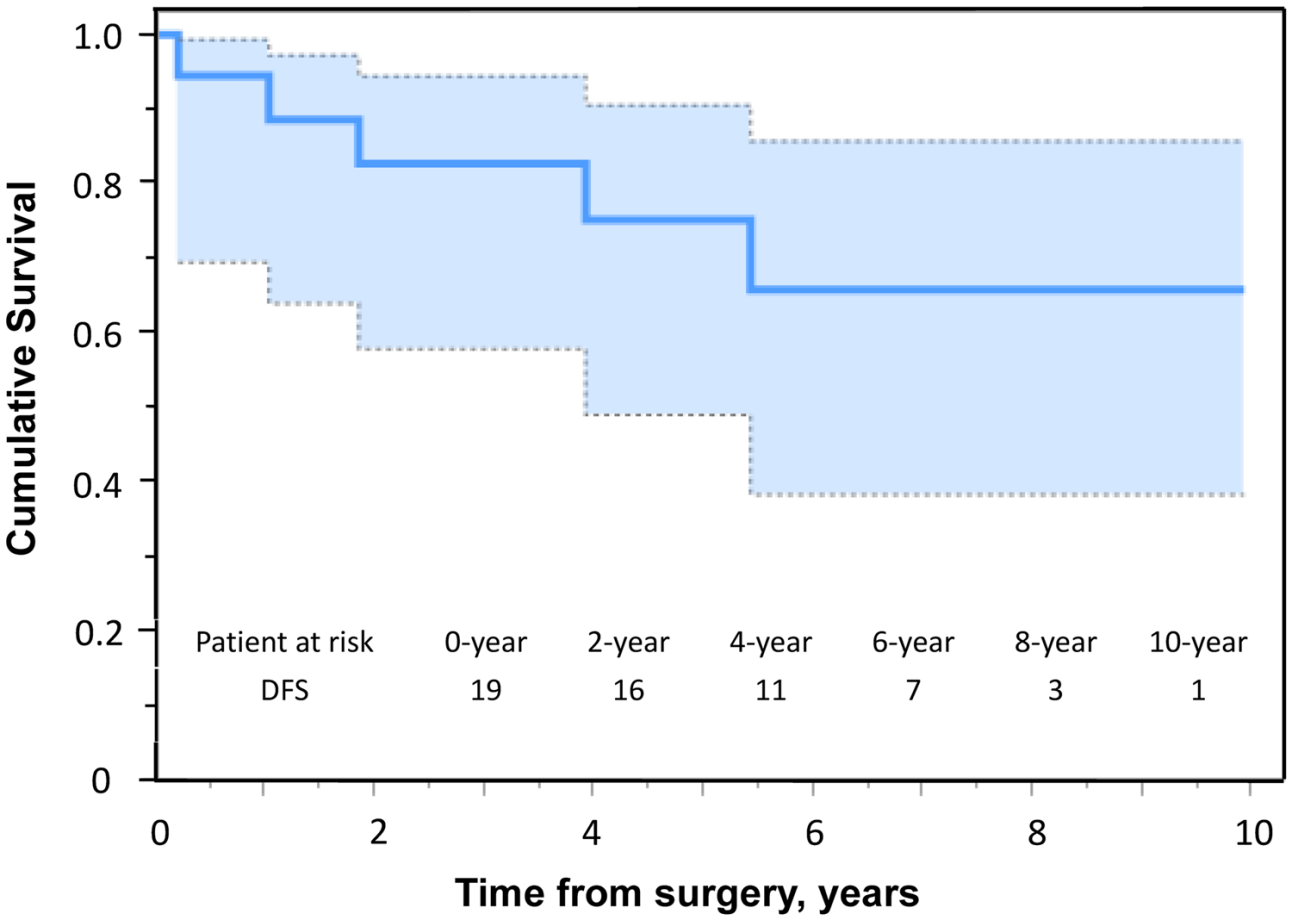
Kaplan–Meier estimates for the disease-free survival curve of 19 patients with primary chest wall sarcoma

Two patients developed distant metastases, and one patient died of her disease 13 months after the operation. The 5-year OS and disease-free survival (DFS) rates were 94% and 68%, respectively (Figs. 2, 3). Local recurrence was observed in three patients, two of whom also had distant metastasis. In the case of isolated local recurrence, the patient received 70.4-Gy proton therapy at the recurrent site and has remained progression-free for two and a half years. One patient with distant metastasis exhibited metastases in the lung, thoracic spine, and mediastinal lymph nodes, and the treatment approach was best supportive care (BSC). The remaining patient had metastasis to the left humerus and received BSC.

### Prognostic factors

**Fig. 4:**
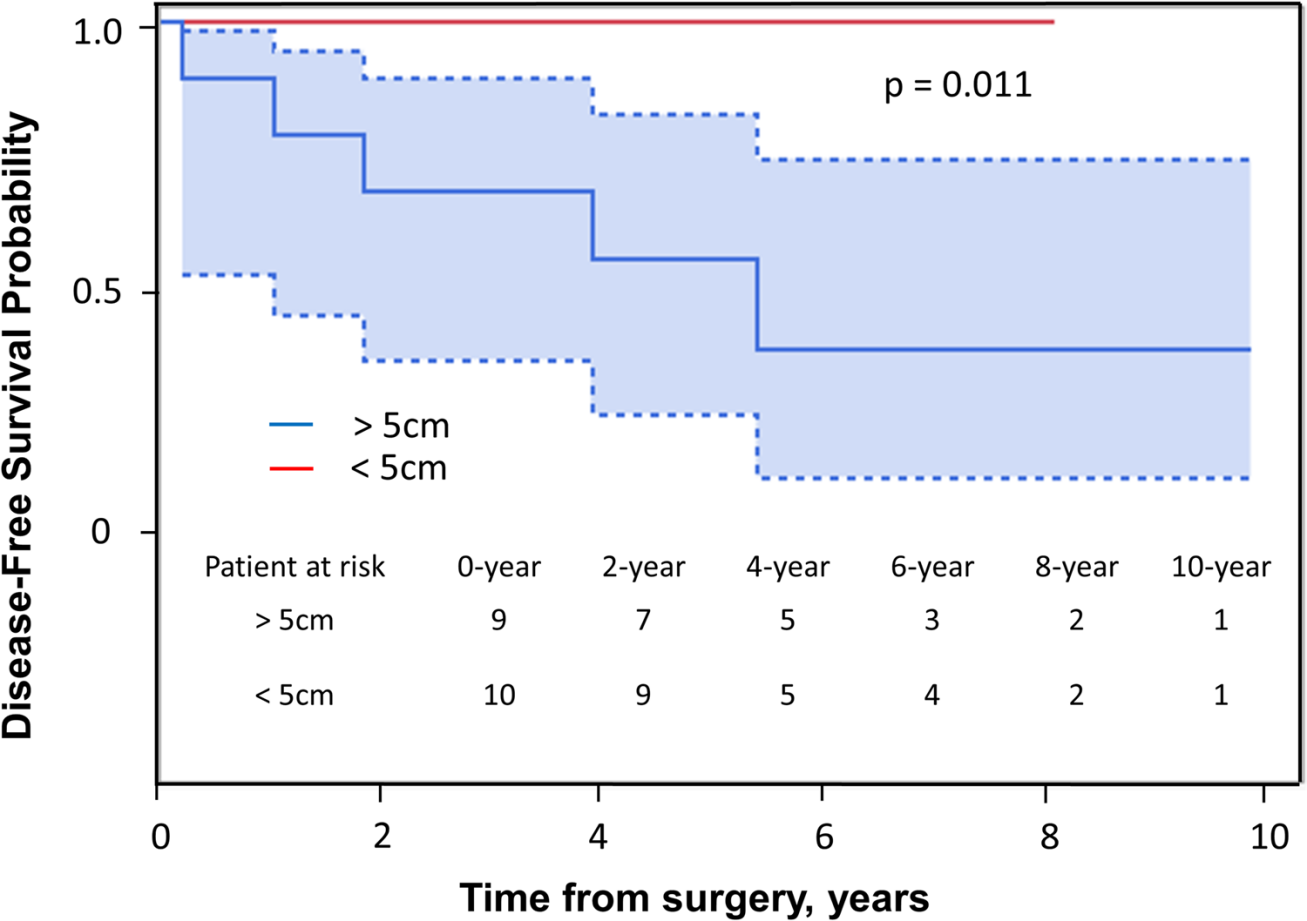
Kaplan–Meier estimates for the disease-free survival curve following primary diagnosis according to tumor size

**Fig. 5:**
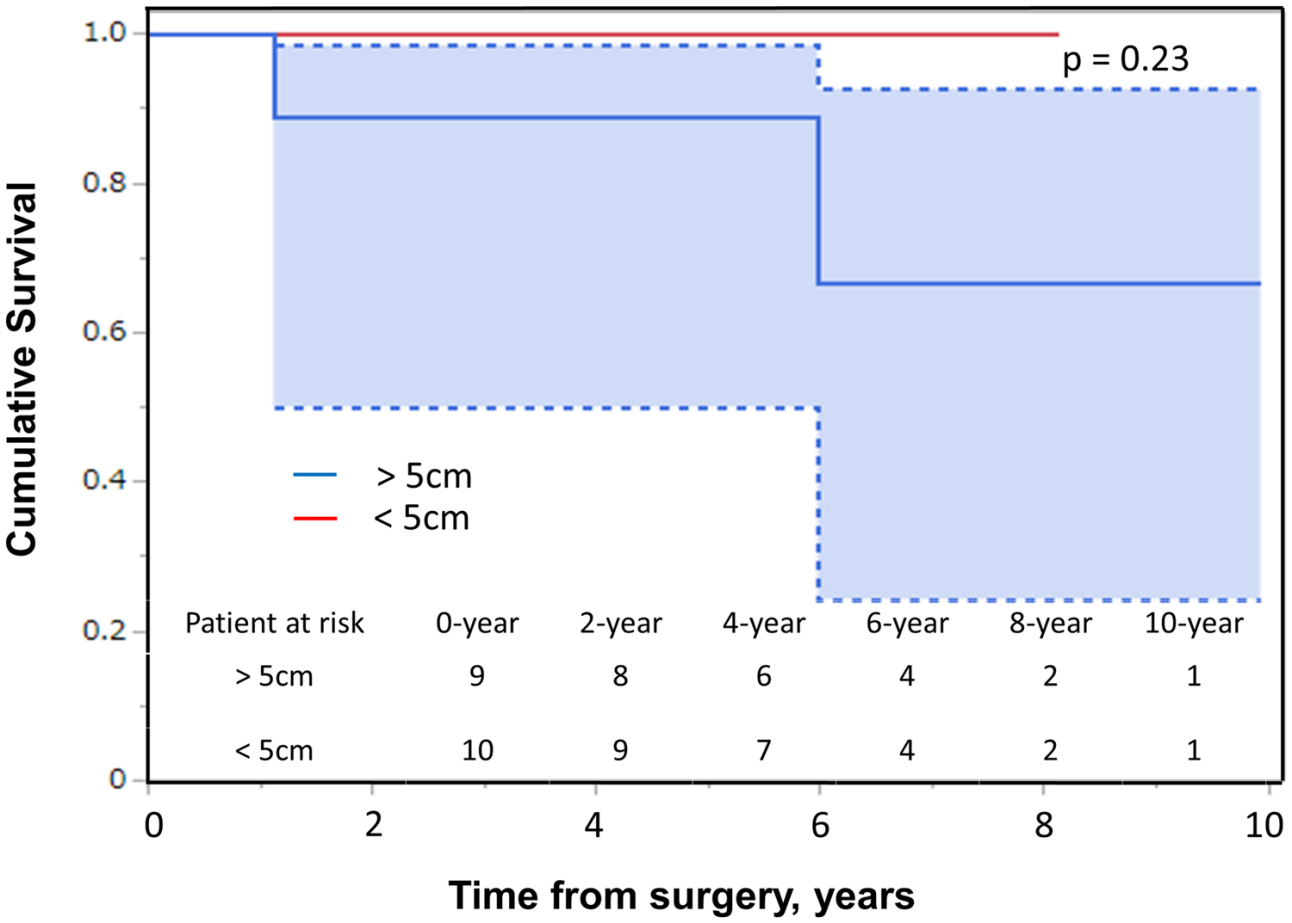
Kaplan–Meier estimates for the overall survival curve following primary diagnosis according to tumor size

In the univariate analyses of DFS, the significant prognostic factors included tumor size (hazard ratio, 1.44; 95% confidence interval, 1.09–2.45; *P* = .011) (Table 3, Fig. 4). However, there was no significant correlation between tumor size and overall survival (OS) (*p* = 0.23) (Fig. 5). In the univariate analyses, age ≥ 70 years (*P* = .027) and tumor size ≥ 5 cm (*P* = .047) were predictors of local recurrence. No factor was found to be a predictor of distant metastases and OS (Table 2).

**Table 3:** Adjusted cox model for disease-free survival

Covariates	HR (95% CI)	*P*-value
Diagnosis (Chondrosarcoma)	5.1 (0.09–279)	0.367
Age	0.98 (0.9–1.06)	0.633
Sex (male)	4.35 (0.47–40.3)	0.164
Grade (2, 3)	2.15 (0.03–163)	0.71
Size	1.44 (1.09–2.45)	0.011
Number of ribs resected	1.25 (0.34–9.93)	0.738

*CI* confidence interval, *HR* hazard ratio

## Discussion

Owing to its rarity, primary chest sarcoma poses challenges in establishing standardized treatment protocols. The scarcity of cases limits studies compared to more common cancers. Managing such rare diseases often requires a multidisciplinary team of specialized healthcare professionals. We analyzed a patient cohort treated for localized primary chest wall sarcoma at our institutions over a 9-year period. However, we acknowledge that different histological subtypes, such as chondrosarcoma and Ewing sarcoma, have distinct biological characteristics, treatment strategies, and prognoses. Due to the limited number of cases in our study, we analyzed all sarcoma types collectively. Future studies with larger cohorts may allow for a more precise stratification of histological subtypes and a better understanding of their respective treatment responses and prognostic factors. Preoperative planning, led by a team of orthopedic, respiratory, and plastic surgeons, consistently achieved R0 resections in all patients. Chest wall reconstructions, often using polypropylene mesh, resulted in early complications for 11% of patients. The 5-year OS and DFS rates stood at 94% and 68%, respectively. Importantly, local recurrences were associated with tumor size ≥ 5 cm and patient age ≥ 70 years.

Resection margins are crucial in sarcoma surgery, yet previous studies on chest wall sarcoma have yielded inconsistent results. Analyzing a larger cohort of 110 patients with chest wall soft tissue sarcoma (STS), univariate analysis showed that R0 resection led to significantly better OS than positive margins. However, the effect on LRFS was not statistically significant [10]. Examining a substantial cohort, McMillan et al. found that 18% of patients underwent incomplete resection, but margin status was not associated with recurrence [11]. In a review involving STS and chondrosarcoma of the chest wall, complete margins did not predict OS or LRFS [12]. Contrastingly, Jitesh et al. reported positive margin status as an independent negative predictor of survival. This underscores the importance of achieving completeness of resection, even in large or advanced tumors, as it remains a primary therapeutic goal [13]. In our study, all patients underwent R0 surgery, potentially contributing to their favorable OS and DFS. Nevertheless, early postoperative complications tend to be less severe compared with other reports of extensive chest wall resection for sarcoma [5, 12, 14–16], with 53% of grade I complications according to CDC classification, but only 16% of grade III or higher complications. This can be attributed to preoperative intervention by the perioperative team and preoperative surgical simulation conducted by multiple departments at our hospital.

For chest wall reconstruction, we used a method that involves the use of two sheets of layered polypropylene mesh, as detailed by Kawana et al. [9]. To prevent local recurrence of sarcoma, a minimum margin of 2 cm is deemed necessary [17]. Defects of < 5 cm in diameter may not need reconstruction [18]. However, larger defects or those over the heart require it to maintain lung function and protect thoracic organs. Chest wall reconstruction with two layered polypropylene mesh sheets offers good stability and flexibility. By stacking two meshes orthogonally, which aligns with polypropylene’s expansion and contraction, rigidity increases. Additionally, polypropylene mesh is easier to trim and more transparent than other rigid materials. Therefore, using two orthogonally stacked polypropylene meshes is an effective, straightforward method for achieving R0 surgery.

Based on prior publications, tumor size and patient age have been recognized as two factors correlated with survival. In a study encompassing 110 chest wall STSs requiring either superficial or full-thickness resection, multivariate analysis emphasized tumor size (> 5 cm vs.  ≤ 5 cm) and patient age (> 50 vs.  ≤ 50 years) as independent prognostic factors influencing OS [10]. In our study, tumor size (> 5 cm vs.  ≤ 5 cm) and patient age (> 70 vs.  ≤ 70 years) were identified as risk factors for local recurrence. Notably, the median patient age in our cohort was 64 years, which is older than the median ages of 45 years and 59.8 years reported in the most frequently cited articles on chest wall sarcomas [10, 13].

Histological tumor grade is a surrogate for cancer biology and has consistently been recognized as the primary predictor of outcomes following chest wall sarcoma resection. In a comprehensive series involving 192 patients with STS of the chest wall, 57% presented with high-grade tumors, and tumor grade emerged as the principal predictor of recurrence. However, no analysis of the association between tumor grade and OS was performed [11]. Moreover, Gross et al., who investigated 55 patients having STS of the chest wall, observed a correlation between grade and DFS and identified it as the sole factor significantly predictive of OS in multivariate analysis [3]. Additionally, Tsukushi et al., in a cohort of 44 patients, found that high tumor grade was associated with mortality on univariate analysis [5]. However, in the present study, the number of grade 1 tumors was significantly small, rendering our analysis underpowered to detect a significant association between tumor grade and survival.

This study has some limitations. Firstly, the rarity of sarcoma led to a small sample size of 19 patients, which constrained our ability to detect factors influencing clinical outcomes. Additionally, some factors investigated did not correlate with complications, but could be significant in a larger study. Secondly, selection bias in administering RT may have occurred; preoperative external beam RT was used when tumors were near major nerves or vessels and achieving a sufficient margin was challenging. Postoperative RT was used for inadequate margins. RT may affect local recurrence rates.

## Conclusion

Wide margin resection and rigid reconstruction are preferred to avert respiratory complications. Chest wall sarcoma is notably aggressive. Vigilant follow-up is crucial, particularly in patients aged ≥ 70 years and those with tumors that are ≥ 5 cm in size, to maintain local control. Larger studies are needed to elucidate the effectiveness of RT and chemotherapy in this uncommon malignancy.

## Data Availability

No datasets were generated or analyzed during the current study.
